# 6α-Hy­droxy-5,6-dihydro­salviasperanol

**DOI:** 10.1107/S160053681004208X

**Published:** 2010-10-23

**Authors:** Safra Izuani Jama Asik, Ibrahim Abdul Razak, Abdul Wahab Salae, Suchada Chantrapromma, Hoong-Kun Fun

**Affiliations:** a X-ray Crystallography Unit, School of Physics, Universiti Sains Malaysia, 11800 USM, Penang, Malaysia; b Crystal Materials Research Unit, Department of Chemistry, Faculty of Science, Prince of Songkla University, Hat-Yai, Songkhla 90112, Thailand

## Abstract

In the title compound, C_20_H_28_O_4_, a diterpenoid isolated from the roots of *Premna obtusifolia* (Verbenaceae), the five-membered ring is in a half-chair conformation. One six-membered ring exists in a twisted-boat conformation while the other is in half-boat conformation. The crystal packing is stabilized by inter­molecular O—H⋯O and weak C—H⋯O inter­actions, generating (001) sheets.

## Related literature

For background to Verbenaceae, diterpenes and their bio­log­ical activity, see: Hymavathi *et al.* (2009 ▶); Bunluepuech & Tewtrakul (2009 ▶); Esquivel *et al.* (1995 ▶). For ring conformations and ring puckering analysis, see: Cremer & Pople (1975 ▶). For bond-length data, see: Allen *et al.* (1987 ▶). For the stability of the temperature controller used in the data collection, see: Cosier & Glazer (1986 ▶).
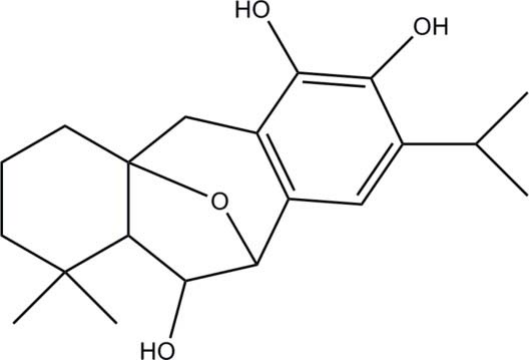


## Experimental

### 

#### Crystal data

C_20_H_28_O_4_
*M* *_r_* = 332.42
Orthorhombic, 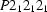

*a* = 6.2767 (2) Å
*b* = 11.7358 (4) Å
*c* = 23.7496 (7) Å
*V* = 1749.45 (10) Å^3^
*Z* = 4
Mo *K*α radiation
μ = 0.09 mm^−1^
*T* = 100 K
0.49 × 0.36 × 0.24 mm

#### Data collection

Bruker SMART APEXII CCD area-detector diffractometer
Absorption correction: multi-scan (*SADABS*; Bruker, 2005) *T*_min_ = 0.959, *T*_max_ = 0.979
15028 measured reflections
3534 independent reflections
3079 reflections with *I* > 2σ(*I*)
*R*_int_ = 0.037

#### Refinement

*R*[*F* ^2^ > 2σ(*F* ^2^)] = 0.041
*wR*(*F* ^2^) = 0.107
*S* = 1.15
3534 reflections
233 parameters
H atoms treated by a mixture of independent and constrained refinement
Δρ_max_ = 0.36 e Å^−3^
Δρ_min_ = −0.25 e Å^−3^

### 

Data collection: *APEX2* (Bruker, 2009 ▶); cell refinement: *SAINT* (Bruker, 2009 ▶); data reduction: *SAINT*; program(s) used to solve structure: *SHELXTL* (Sheldrick, 2008 ▶); program(s) used to refine structure: *SHELXTL*; molecular graphics: *SHELXTL*; software used to prepare material for publication: *SHELXTL* and *PLATON* (Spek, 2009 ▶).

## Supplementary Material

Crystal structure: contains datablocks global, I. DOI: 10.1107/S160053681004208X/hb5689sup1.cif

Structure factors: contains datablocks I. DOI: 10.1107/S160053681004208X/hb5689Isup2.hkl

Additional supplementary materials:  crystallographic information; 3D view; checkCIF report

## Figures and Tables

**Table 1:** Hydrogen-bond geometry (Å, °)

*D*—H⋯*A*	*D*—H	H⋯*A*	*D*⋯*A*	*D*—H⋯*A*
O3—H1*O*3⋯O2^i^	0.85 (3)	2.01 (3)	2.8504 (17)	169 (3)
O4—H1*O*4⋯O1^ii^	0.84 (3)	1.89 (3)	2.7089 (16)	165 (3)
C18—H18*B*⋯O3^iii^	0.96	2.55	3.407 (2)	149

Symmetry codes: (i) 
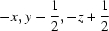
; (ii) 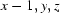; (iii) 
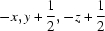
.

**Fig. 1.**
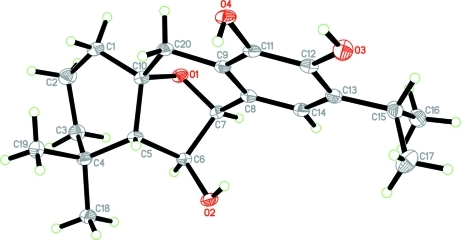
The structure of (I), showing 50% probability displacement ellipsoids.

**Fig. 2.**
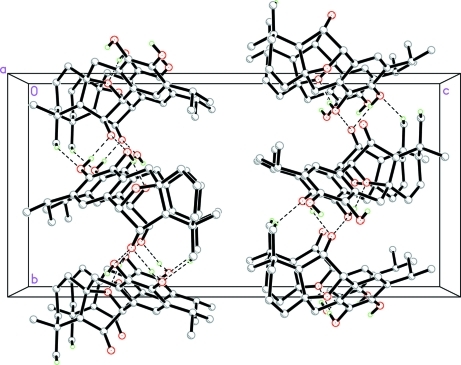
The crystal packing of (I) viewed along the a axis, showing infinite two dimensional networks parallel to ab plane. Hydrogen bonds are shown as dashed lines.

## supplementary crystallographic information

### Comment

Plants of the family Verbenaceae were found to possess interesting biological properties such as cytotoxicity (Hymavathi *et al.*, 2009) and anti-HIV-1 integrase activities (Bunluepuech & Tewtrakul, 2009). The phytochemistry study of the aerial parts of *Premna obtusifolia* (Verbenaceae), a small tree found in the mangrove forests which were collected from Satun province in the southern part of Thailand, led to the isolation of diterpenes. The title compound which is known as 5,6-dihydro-6α-hydroxysalviasperanol (Esquivel *et al.*, 1995) is one of the isolated compounds from this plant. Herein we report the crystal structure of the title compound (I).

The bond lengths show normal values (Allen *et al.*, 1987). The pyrocatechol, C8/C9/C11–C14/O3/O4, is planar with the maximum deviation of 0.006Å for atom C12. The five-membered ring, C5–C7/C10/O1, is in half-chair conformation with the puckering parameter Q = 0.4588 (16)Å, φ = 194.0 (2)°. The six-membered ring, C1–C5/C10 adopts twisted-boat conformation with puckering parameter Q = 0.6536 (18)Å, θ = 79.56 (16)° and φ = 156.60 (16)°. The other six-membered ring , C7–C10/C20/O1, is in half-boat conformation with puckering parameter Q = 0.5929 (15)Å, θ = 47.83 (15)° and φ = 347.9 (2)° (Cremer & Pople 1975). The torsion angles of propanyl group attached to the pyrocatechol ring are C14–C13–C15–C17 = -101.80 (19)° and C14–C13–C15–C16 = 78.55 (19)°.

The crystal packing of (I) is stabilized by intermolecular O3—H1O3···O2 and O4—H1O4···O1 and weak C18—H18B···O3 interactions. The molecules are linked into infinite two dimensional networks parallel to *ab* plane.

### Experimental

The air-dried roots of *Premna obtusifolia* (4.5 kg) were extracted with CH_2_Cl_2_ (2 *x* 20 *L*) under room temperature. The combined extracts were concentrated under reduced pressure to give a dark yellow extract (40.5g) which was subjected to quick column chromatography (QCC) over silicagel using solvents of increasing polarity from n–hexane to EtOAc to afford 12 fractions (F1–F12). Fraction F10 was further purified by QCC using CH_2_Cl_2_–EtOAc (3:7), yielding compound (I) (145.8 mg). Colorless block-shaped single crystals of (I) were recrystallized from CH_2_Cl_2_ after several days (m.p.461–463 K).

### Refinement

Anomalous dispersion was negligible and Friedel pairs were merged before refinement. O bound H atoms were located  from the difference map and isotropically refined.The remaining H atoms were placed in calculated positions with (C—H) = 0.98 for CH, 0.97 for CH_2_, 0.96  for CH_3_ and 0.93 Å for CH in benzene group. The *U*_iso_ values were constrained to be 1.5*U*_eq_ of the carrier atom for methyl H atoms and 1.2*U*_eq_ for the remaining H atoms. A rotating group model was used for the methyl groups.

### Crystal data

**Table:**

C_20_H_28_O_4_	*F*(000) = 720
*M**_r_* = 332.42	*D*_x_ = 1.262 Mg m^−^^3^
Orthorhombic, *P*2_1_2_1_2_1_	Mo *K*α radiation, λ = 0.71073 Å
Hall symbol: P 2ac 2ab	Cell parameters from 6222 reflections
*a* = 6.2767 (2) Å	θ = 2.4–32.3°
*b* = 11.7358 (4) Å	µ = 0.09 mm^−^^1^
*c* = 23.7496 (7) Å	*T* = 100 K
*V* = 1749.45 (10) Å^3^	Block, colourless
*Z* = 4	0.49 × 0.36 × 0.24 mm

### Data collection

**Table:**

Bruker SMART APEXII CCD area-detector diffractometer	3534 independent reflections
Radiation source: sealed tube	3079 reflections with *I* > 2σ(*I*)
graphite	*R*_int_ = 0.037
φ and ω scans	θ_max_ = 32.4°, θ_min_ = 2.4°
Absorption correction: multi-scan (*SADABS*; Bruker, 2005)	*h* = −8→9
*T*_min_ = 0.959, *T*_max_ = 0.979	*k* = −17→16
15028 measured reflections	*l* = −34→35

### Refinement

**Table:**

Refinement on *F*^2^	Primary atom site location: structure-invariant direct methods
Least-squares matrix: full	Secondary atom site location: difference Fourier map
*R*[*F*^2^ > 2σ(*F*^2^)] = 0.041	Hydrogen site location: inferred from neighbouring sites
*wR*(*F*^2^) = 0.107	H atoms treated by a mixture of independent and constrained refinement
*S* = 1.15	*w* = 1/[σ^2^(*F*_o_^2^) + (0.0548*P*)^2^ + 0.2019*P*] where *P* = (*F*_o_^2^ + 2*F*_c_^2^)/3
3534 reflections	(Δ/σ)_max_ < 0.001
233 parameters	Δρ_max_ = 0.36 e Å^−^^3^
0 restraints	Δρ_min_ = −0.25 e Å^−^^3^

### Special details

**Table:**

Experimental. The crystal was placed in the cold stream of an Oxford Cryosystems Cobra open-flow nitrogen cryostat (Cosier & Glazer, 1986) operating at 100.0 (1) K.
Geometry. All esds (except the esd in the dihedral angle between two l.s. planes) are estimated using the full covariance matrix. The cell esds are taken into account individually in the estimation of esds in distances, angles and torsion angles; correlations between esds in cell parameters are only used when they are defined by crystal symmetry. An approximate (isotropic) treatment of cell esds is used for estimating esds involving l.s. planes.
Refinement. Refinement of F^2^ against ALL reflections. The weighted R-factor wR and goodness of fit S are based on F^2^, conventional R-factors R are based on F, with F set to zero for negative F^2^. The threshold expression of F^2^ > 2sigma(F^2^) is used only for calculating R-factors(gt) etc. and is not relevant to the choice of reflections for refinement. R-factors based on F^2^ are statistically about twice as large as those based on F, and R- factors based on ALL data will be even larger.

### Fractional atomic coordinates and isotropic or equivalent isotropic displacement parameters (Å^2^)

**Table:**

	*x*	*y*	*z*	*U*_iso_*/*U*_eq_	
O1	0.7000 (2)	0.51248 (9)	0.29871 (5)	0.0162 (2)	
O2	0.3927 (2)	0.76867 (10)	0.28241 (5)	0.0179 (2)	
H1O2	0.295 (4)	0.730 (2)	0.2677 (10)	0.035 (7)*	
O3	−0.1349 (2)	0.42654 (11)	0.15694 (5)	0.0212 (3)	
H1O3	−0.199 (5)	0.379 (2)	0.1782 (10)	0.036 (7)*	
O4	−0.0204 (2)	0.35135 (9)	0.26393 (5)	0.0175 (2)	
H1O4	−0.122 (5)	0.391 (2)	0.2762 (10)	0.034 (7)*	
C1	0.6494 (3)	0.43356 (13)	0.39056 (7)	0.0193 (3)	
H1A	0.7202	0.3676	0.3746	0.023*	
H1B	0.5407	0.4058	0.4162	0.023*	
C2	0.8142 (3)	0.50212 (15)	0.42483 (7)	0.0231 (3)	
H2A	0.8018	0.4811	0.4642	0.028*	
H2B	0.9560	0.4811	0.4124	0.028*	
C3	0.7889 (3)	0.63252 (15)	0.41961 (7)	0.0200 (3)	
H3A	0.8483	0.6684	0.4529	0.024*	
H3B	0.8699	0.6588	0.3873	0.024*	
C4	0.5563 (3)	0.67027 (13)	0.41290 (6)	0.0162 (3)	
C5	0.4707 (3)	0.62326 (12)	0.35629 (6)	0.0141 (3)	
H5A	0.3147	0.6257	0.3574	0.017*	
C6	0.5467 (3)	0.69065 (12)	0.30370 (6)	0.0141 (3)	
H6A	0.6753	0.7334	0.3138	0.017*	
C7	0.6093 (3)	0.59624 (12)	0.26148 (6)	0.0146 (3)	
H7A	0.7152	0.6239	0.2344	0.018*	
C8	0.4169 (3)	0.54859 (12)	0.23181 (6)	0.0145 (3)	
C9	0.2902 (3)	0.47081 (12)	0.26130 (6)	0.0144 (3)	
C10	0.5406 (3)	0.49841 (12)	0.34320 (6)	0.0147 (3)	
C11	0.1059 (3)	0.43033 (12)	0.23603 (6)	0.0145 (3)	
C12	0.0464 (3)	0.46583 (13)	0.18190 (6)	0.0155 (3)	
C13	0.1739 (3)	0.54262 (13)	0.15161 (6)	0.0161 (3)	
C14	0.3583 (3)	0.58331 (12)	0.17780 (6)	0.0154 (3)	
H14A	0.4443	0.6350	0.1587	0.018*	
C15	0.1063 (3)	0.57665 (14)	0.09244 (6)	0.0196 (3)	
H15A	0.0468	0.5085	0.0745	0.023*	
C16	0.2922 (3)	0.61702 (16)	0.05555 (7)	0.0237 (4)	
H16A	0.2431	0.6281	0.0177	0.036*	
H16B	0.4030	0.5606	0.0559	0.036*	
H16C	0.3468	0.6876	0.0700	0.036*	
C17	−0.0714 (3)	0.66608 (18)	0.09427 (8)	0.0286 (4)	
H17A	−0.1905	0.6364	0.1150	0.043*	
H17B	−0.1153	0.6841	0.0566	0.043*	
H17C	−0.0197	0.7338	0.1124	0.043*	
C18	0.5432 (3)	0.80127 (14)	0.41448 (7)	0.0205 (3)	
H18A	0.5862	0.8280	0.4510	0.031*	
H18B	0.3994	0.8248	0.4072	0.031*	
H18C	0.6359	0.8326	0.3863	0.031*	
C19	0.4188 (3)	0.62608 (15)	0.46181 (6)	0.0213 (3)	
H19A	0.4798	0.6501	0.4969	0.032*	
H19B	0.4136	0.5444	0.4606	0.032*	
H19C	0.2771	0.6561	0.4585	0.032*	
C20	0.3572 (3)	0.42821 (12)	0.31863 (6)	0.0158 (3)	
H20A	0.4012	0.3492	0.3156	0.019*	
H20B	0.2363	0.4316	0.3440	0.019*	

### Atomic displacement parameters (Å^2^)

**Table:**

	*U*^11^	*U*^22^	*U*^33^	*U*^12^	*U*^13^	*U*^23^
O1	0.0152 (6)	0.0138 (5)	0.0197 (5)	0.0030 (4)	0.0026 (4)	0.0022 (4)
O2	0.0206 (6)	0.0119 (5)	0.0213 (5)	0.0033 (5)	−0.0016 (5)	0.0015 (4)
O3	0.0204 (7)	0.0206 (5)	0.0226 (5)	−0.0081 (5)	−0.0026 (5)	0.0026 (4)
O4	0.0182 (6)	0.0109 (5)	0.0234 (5)	−0.0024 (4)	0.0037 (5)	0.0006 (4)
C1	0.0231 (9)	0.0138 (6)	0.0211 (6)	0.0039 (6)	−0.0016 (6)	0.0029 (5)
C2	0.0221 (9)	0.0215 (8)	0.0257 (7)	0.0055 (7)	−0.0053 (7)	0.0015 (6)
C3	0.0194 (8)	0.0193 (7)	0.0214 (7)	−0.0001 (7)	−0.0029 (6)	−0.0001 (6)
C4	0.0188 (8)	0.0126 (6)	0.0173 (6)	−0.0004 (6)	−0.0016 (6)	0.0002 (5)
C5	0.0145 (7)	0.0107 (6)	0.0171 (6)	0.0005 (6)	0.0002 (5)	0.0004 (5)
C6	0.0144 (7)	0.0111 (6)	0.0168 (6)	0.0006 (6)	−0.0005 (5)	−0.0001 (5)
C7	0.0153 (7)	0.0120 (6)	0.0165 (6)	0.0000 (6)	0.0008 (5)	0.0015 (5)
C8	0.0147 (8)	0.0115 (6)	0.0174 (6)	0.0003 (5)	0.0014 (5)	−0.0010 (5)
C9	0.0162 (7)	0.0102 (6)	0.0168 (6)	0.0009 (5)	0.0023 (6)	−0.0011 (5)
C10	0.0139 (7)	0.0122 (6)	0.0180 (6)	0.0007 (6)	0.0007 (5)	0.0013 (5)
C11	0.0161 (7)	0.0086 (5)	0.0189 (6)	−0.0002 (5)	0.0037 (5)	−0.0002 (5)
C12	0.0146 (8)	0.0120 (6)	0.0198 (6)	−0.0009 (6)	0.0003 (6)	−0.0019 (5)
C13	0.0184 (8)	0.0130 (6)	0.0168 (6)	0.0008 (6)	0.0011 (6)	−0.0012 (5)
C14	0.0169 (8)	0.0118 (6)	0.0174 (6)	−0.0009 (6)	0.0027 (5)	−0.0009 (5)
C15	0.0236 (9)	0.0180 (7)	0.0171 (6)	−0.0057 (7)	−0.0017 (6)	0.0002 (5)
C16	0.0324 (10)	0.0202 (7)	0.0186 (6)	−0.0058 (8)	0.0027 (7)	−0.0002 (6)
C17	0.0235 (10)	0.0304 (9)	0.0318 (8)	0.0020 (8)	−0.0039 (7)	0.0089 (7)
C18	0.0269 (9)	0.0144 (6)	0.0203 (6)	0.0009 (7)	−0.0026 (7)	−0.0020 (5)
C19	0.0262 (9)	0.0206 (7)	0.0171 (6)	0.0005 (7)	0.0028 (6)	0.0013 (6)
C20	0.0193 (8)	0.0100 (6)	0.0181 (6)	−0.0015 (6)	0.0011 (6)	0.0006 (5)

### Geometric parameters (Å, °)

**Table:**

O1—C7	1.4395 (18)	C8—C14	1.395 (2)
O1—C10	1.4647 (19)	C8—C9	1.399 (2)
O2—C6	1.4244 (19)	C9—C11	1.387 (2)
O2—H1O2	0.84 (3)	C9—C20	1.510 (2)
O3—C12	1.363 (2)	C10—C20	1.531 (2)
O3—H1O3	0.85 (3)	C11—C12	1.402 (2)
O4—C11	1.3882 (18)	C12—C13	1.404 (2)
O4—H1O4	0.84 (3)	C13—C14	1.398 (2)
C1—C10	1.520 (2)	C13—C15	1.521 (2)
C1—C2	1.543 (3)	C14—H14A	0.9300
C1—H1A	0.9700	C15—C17	1.532 (3)
C1—H1B	0.9700	C15—C16	1.534 (2)
C2—C3	1.544 (2)	C15—H15A	0.9800
C2—H2A	0.9700	C16—H16A	0.9600
C2—H2B	0.9700	C16—H16B	0.9600
C3—C4	1.535 (3)	C16—H16C	0.9600
C3—H3A	0.9700	C17—H17A	0.9600
C3—H3B	0.9700	C17—H17B	0.9600
C4—C19	1.537 (2)	C17—H17C	0.9600
C4—C18	1.540 (2)	C18—H18A	0.9600
C4—C5	1.549 (2)	C18—H18B	0.9600
C5—C6	1.553 (2)	C18—H18C	0.9600
C5—C10	1.561 (2)	C19—H19A	0.9600
C5—H5A	0.9800	C19—H19B	0.9600
C6—C7	1.545 (2)	C19—H19C	0.9600
C6—H6A	0.9800	C20—H20A	0.9700
C7—C8	1.506 (2)	C20—H20B	0.9700
C7—H7A	0.9800		
			
C7—O1—C10	104.47 (12)	O1—C10—C20	107.42 (12)
C6—O2—H1O2	107.5 (18)	C1—C10—C20	110.51 (12)
C12—O3—H1O3	110.8 (18)	O1—C10—C5	103.28 (11)
C11—O4—H1O4	103.5 (18)	C1—C10—C5	116.68 (12)
C10—C1—C2	115.50 (13)	C20—C10—C5	111.72 (13)
C10—C1—H1A	108.4	C9—C11—O4	119.91 (13)
C2—C1—H1A	108.4	C9—C11—C12	121.15 (14)
C10—C1—H1B	108.4	O4—C11—C12	118.93 (14)
C2—C1—H1B	108.4	O3—C12—C11	121.38 (14)
H1A—C1—H1B	107.5	O3—C12—C13	118.05 (13)
C3—C2—C1	113.94 (14)	C11—C12—C13	120.57 (15)
C3—C2—H2A	108.8	C14—C13—C12	117.61 (14)
C1—C2—H2A	108.8	C14—C13—C15	123.51 (14)
C3—C2—H2B	108.8	C12—C13—C15	118.88 (15)
C1—C2—H2B	108.8	C8—C14—C13	121.84 (14)
H2A—C2—H2B	107.7	C8—C14—H14A	119.1
C4—C3—C2	113.11 (15)	C13—C14—H14A	119.1
C4—C3—H3A	109.0	C13—C15—C17	110.89 (14)
C2—C3—H3A	109.0	C13—C15—C16	113.37 (15)
C4—C3—H3B	109.0	C17—C15—C16	111.00 (15)
C2—C3—H3B	109.0	C13—C15—H15A	107.1
H3A—C3—H3B	107.8	C17—C15—H15A	107.1
C3—C4—C19	111.00 (13)	C16—C15—H15A	107.1
C3—C4—C18	109.66 (15)	C15—C16—H16A	109.5
C19—C4—C18	106.77 (14)	C15—C16—H16B	109.5
C3—C4—C5	108.49 (13)	H16A—C16—H16B	109.5
C19—C4—C5	109.94 (14)	C15—C16—H16C	109.5
C18—C4—C5	110.99 (13)	H16A—C16—H16C	109.5
C4—C5—C6	114.20 (12)	H16B—C16—H16C	109.5
C4—C5—C10	114.19 (13)	C15—C17—H17A	109.5
C6—C5—C10	103.40 (11)	C15—C17—H17B	109.5
C4—C5—H5A	108.3	H17A—C17—H17B	109.5
C6—C5—H5A	108.3	C15—C17—H17C	109.5
C10—C5—H5A	108.3	H17A—C17—H17C	109.5
O2—C6—C7	113.77 (12)	H17B—C17—H17C	109.5
O2—C6—C5	113.85 (13)	C4—C18—H18A	109.5
C7—C6—C5	103.57 (11)	C4—C18—H18B	109.5
O2—C6—H6A	108.5	H18A—C18—H18B	109.5
C7—C6—H6A	108.5	C4—C18—H18C	109.5
C5—C6—H6A	108.5	H18A—C18—H18C	109.5
O1—C7—C8	110.55 (12)	H18B—C18—H18C	109.5
O1—C7—C6	101.05 (11)	C4—C19—H19A	109.5
C8—C7—C6	111.48 (13)	C4—C19—H19B	109.5
O1—C7—H7A	111.1	H19A—C19—H19B	109.5
C8—C7—H7A	111.1	C4—C19—H19C	109.5
C6—C7—H7A	111.1	H19A—C19—H19C	109.5
C14—C8—C9	120.06 (15)	H19B—C19—H19C	109.5
C14—C8—C7	122.22 (13)	C9—C20—C10	112.02 (12)
C9—C8—C7	117.66 (13)	C9—C20—H20A	109.2
C11—C9—C8	118.76 (14)	C10—C20—H20A	109.2
C11—C9—C20	120.60 (14)	C9—C20—H20B	109.2
C8—C9—C20	120.59 (14)	C10—C20—H20B	109.2
O1—C10—C1	106.44 (13)	H20A—C20—H20B	107.9
			
C10—C1—C2—C3	19.7 (2)	C2—C1—C10—C20	−171.02 (14)
C1—C2—C3—C4	33.4 (2)	C2—C1—C10—C5	−42.0 (2)
C2—C3—C4—C19	56.16 (18)	C4—C5—C10—O1	−106.69 (14)
C2—C3—C4—C18	173.89 (13)	C6—C5—C10—O1	17.99 (15)
C2—C3—C4—C5	−64.74 (16)	C4—C5—C10—C1	9.7 (2)
C3—C4—C5—C6	−77.19 (16)	C6—C5—C10—C1	134.36 (14)
C19—C4—C5—C6	161.26 (14)	C4—C5—C10—C20	138.16 (14)
C18—C4—C5—C6	43.4 (2)	C6—C5—C10—C20	−97.16 (14)
C3—C4—C5—C10	41.52 (17)	C8—C9—C11—O4	178.72 (13)
C19—C4—C5—C10	−80.04 (17)	C20—C9—C11—O4	1.1 (2)
C18—C4—C5—C10	162.07 (14)	C8—C9—C11—C12	0.2 (2)
C4—C5—C6—O2	−100.27 (16)	C20—C9—C11—C12	−177.48 (14)
C10—C5—C6—O2	135.06 (13)	C9—C11—C12—O3	−179.81 (14)
C4—C5—C6—C7	135.67 (14)	O4—C11—C12—O3	1.6 (2)
C10—C5—C6—C7	11.00 (16)	C9—C11—C12—C13	0.7 (2)
C10—O1—C7—C8	−67.98 (14)	O4—C11—C12—C13	−177.83 (14)
C10—O1—C7—C6	50.16 (14)	O3—C12—C13—C14	179.36 (14)
O2—C6—C7—O1	−160.80 (13)	C11—C12—C13—C14	−1.2 (2)
C5—C6—C7—O1	−36.69 (15)	O3—C12—C13—C15	−1.0 (2)
O2—C6—C7—C8	−43.34 (17)	C11—C12—C13—C15	178.49 (14)
C5—C6—C7—C8	80.77 (14)	C9—C8—C14—C13	0.1 (2)
O1—C7—C8—C14	−149.31 (14)	C7—C8—C14—C13	−176.94 (14)
C6—C7—C8—C14	99.13 (16)	C12—C13—C14—C8	0.8 (2)
O1—C7—C8—C9	33.56 (18)	C15—C13—C14—C8	−178.89 (15)
C6—C7—C8—C9	−77.99 (16)	C14—C13—C15—C17	−101.80 (19)
C14—C8—C9—C11	−0.6 (2)	C12—C13—C15—C17	78.55 (19)
C7—C8—C9—C11	176.60 (13)	C14—C13—C15—C16	23.9 (2)
C14—C8—C9—C20	177.05 (14)	C12—C13—C15—C16	−155.80 (15)
C7—C8—C9—C20	−5.8 (2)	C11—C9—C20—C10	−169.78 (14)
C7—O1—C10—C1	−166.58 (12)	C8—C9—C20—C10	12.6 (2)
C7—O1—C10—C20	75.03 (13)	O1—C10—C20—C9	−46.44 (16)
C7—O1—C10—C5	−43.17 (14)	C1—C10—C20—C9	−162.17 (13)
C2—C1—C10—O1	72.64 (16)	C5—C10—C20—C9	66.15 (16)

### Hydrogen-bond geometry (Å, °)

**Table:**

*D*—H···*A*	*D*—H	H···*A*	*D*···*A*	*D*—H···*A*
O3—H1O3···O2^i^	0.85 (3)	2.01 (3)	2.8504 (17)	169 (3)
O4—H1O4···O1^ii^	0.84 (3)	1.89 (3)	2.7089 (16)	165 (3)
C18—H18B···O3^iii^	0.96	2.55	3.407 (2)	149

Symmetry codes: (i) −*x*, *y*−1/2, −*z*+1/2; (ii) *x*−1, *y*, *z*; (iii) −*x*, *y*+1/2, −*z*+1/2.
